# Developing and evaluating stereotactic lung RT trials: what we should know about the influence of inhomogeneity corrections on dose

**DOI:** 10.1186/1748-717X-3-21

**Published:** 2008-07-28

**Authors:** Danny Schuring, Coen W Hurkmans

**Affiliations:** 1 Catharina-hospital, Department of radiotherapy, Michelangelolaan 2, P.O box 1350, 5602 ZA, Eindhoven, The Netherlands

## Abstract

**Purpose:**

To investigate the influence of inhomogeneity corrections on stereotactic treatment plans for non-small cell lung cancer and determine the dose delivered to the PTV and OARs.

**Materials and methods:**

For 26 patients with stage-I NSCLC treatment plans were optimized with unit density (UD), an equivalent pathlength algorithm (EPL), and a collapsed-cone (CC) algorithm, prescribing 60 Gy to the PTV. After optimization the first two plans were recalculated with the more accurate CC algorithm. Dose parameters were compared for the three different optimized plans. Dose to the target and OARs was evaluated for the recalculated plans and compared with the planned values.

**Results:**

For the CC algorithm dose constraints for the ratio of the 50% isodose volume and the PTV, and the V_20 Gy _are harder to fulfill. After recalculation of the UD and EPL plans large variations in the dose to the PTV were observed. For the unit density plans, the dose to the PTV varied from 42.1 to 63.4 Gy for individual patients. The EPL plans all overestimated the PTV dose (average 48.0 Gy). For the lungs, the recalculated V_20 Gy _was highly correlated to the planned value, and was 12% higher for the UD plans (R^2 ^= 0.99), and 15% lower for the EPL plans (R^2 ^= 0.96).

**Conclusion:**

Inhomogeneity corrections have a large influence on the dose delivered to the PTV and OARs for SBRT of lung tumors. A simple rescaling of the dose to the PTV is not possible, implicating that accurate dose calculations are necessary for these treatment plans in order to prevent large discrepancies between planned and actually delivered doses to individual patients.

## Introduction

Treatment outcome of conventional radiotherapy for early-stage lung cancer has been rather poor, while possibilities for dose escalation are limited. In recent years several studies have shown promising results using stereotactic body radiotherapy (SBRT) for lung tumors, with local control rates at 3 years up to 90% [1-3].

A wide variety of treatment planning algorithms is used for SBRT. As a result, large differences exist in the way that inhomogeneities in the target volume are handled in the planning phase. In two important SBRT of lung cancer trials, on which many current clinical implementations of SBRT are based, different algorithms were used; the RTOG 0236 phase-II trial planning was performed without using inhomogeneity corrections assuming the patient has unit density [4], while in the Japanese JCOG 0403 trial a wide variety of inhomogeneity correction algorithms were allowed [5].

Planning algorithms can roughly be separated in (a) ones which do not take into account changes in lateral electron transport (pencil beam-like algorithms) and (b) ones that do take into account these changes (convolution-superposition type algorithms) [6]. In the type-a algorithms the effects of inhomogeneities are accounted for by applying a correction based on equivalent pathlength (EPL), like the Batho or ETAR correction. In the type-b algorithms changes in the lateral transport are modeled in an approximate way, and several studies have shown these algorithms to be more accurate for dose calculations in regions with inhomogeneities [7,5,8]. In particular, the collapsed-cone convolution-superposition algorithm in most cases shows satisfactory agreement with Monte Carlo simulations in the case of inhomogeneous targets [9,10]. The Monte Carlo algorithms can be seen as the current gold standard for these types of dose calculations.

Several authors have studied the influence of inhomogeneity corrections on dose distributions specifically for stereotactic treatments of lung cancer [7,11-14]. Compared to conventional radiotherapy larger deviations can be expected due to the small field sizes used for treating these tumors. Most studies concentrated on creating a treatment plan using a type-a pencil beam algorithm, and recalculating the plans with a type-b algorithm or using Monte Carlo simulations, mostly for a small number of patients with relatively large tumors or on a phantom. All these studies have shown a significant overestimation of the target dose when using pencil-beam calculations.

In this study, the influence of inhomogeneity corrections on the dose distributions was investigated for a large group of patients with small stage I lung cancer tumors. These results are e.g., important for the correct interpretation of previous clinical trials and for the definition of planning criteria for new clinical trials of this treatment, and are being used for designing a Dutch multicenter randomized phase-III trial comparing SBRT with surgery for stage-I NSCLC (ROSEL trial).

## Materials and methods

### Respiration-correlated CT and target delineation

All patients in this study received a respiration-correlated 4D-CT using a Philips Brilliance Big Bore CT prior to treatment. The 4D-CT was reconstructed in ten equally spaced time bins using phase binning. From these phases, a maximum intensity projection (MIP) was reconstructed. The datasets were then imported in the Pinnacle^3 ^treatment planning system (Philips Medical Systems, Wisconsin). Using the MIP dataset, an experienced radiation oncologist delineated the internal target volume (ITV). The GTV was delineated on the CT dataset of the maximum inhale phase; tumor mobility was determined by translating the delineated GTV from this phase to the maximum exhale phase. Organs at risk were delineated on an average-density CT reconstruction. As dictated by the RTOG 0236 protocol, no ITV to CTV margin was applied [4]. The PTV was created by expanding the ITV with a 3 mm margin to account for setup uncertainties in accordance with the protocol used in by Lagerwaard *et al *[3].

### Patient characteristics

Twentysix consecutive patients with non-small cell lung cancer (NSCLC) were included. All patients had solitary stage-I tumors, were medically inoperable and were treated at our institute with SBRT. A summary of the volumetric and motion characteristics of these tumors is shown in Table 1. The median, 25%, 50%, 75% and 100% percentile values for the PTV were 29.4, 15.8, 29.4, 40.2, and 107.6 cm^3^, respectively.

**Table 1:** Patient characteristics of the group of 26 patients used in this study.

**Characteristics**	**Mean value (range)**
GTV (cm^3^)	13.9 [0.7–50.4]
ITV (cm^3^)	19.3 [1.4–64.1]
PTV (cm^3^)	37.3 [5.5–107.6]
V_ITV_/V_GTV_	1.6 [1.0–2.7]
Peak-to-peak amplitude (cm)	0.8 [0.0–3.0]
Lung volume (cm^3^)	4942 [2720–8786]

### Treatment planning

The treatment plans consisted of 9 equally spaced coplanar 6 MV beams. Beams consisting of 2 segments were not allowed to enter through the esophagus, heart, spinal cord or contralateral lung. The plans were inversely optimized using the direct aperture optimization module of the Pinnacle^3 ^treatment planning system. Dose calculations were performed on an average-density CT using a 3 × 3 × 3 cm dose calculation grid size.

Plans were optimized until 95% of the PTV received the prescription dose of 60 Gy in 3 fractions (according to RTOG 0236), and more than 99% of the PTV received 90% of the prescribed dose (54 Gy). No limitations to the maximum dose were applied within the PTV as highly inhomogeneous dose distributions are commonly accepted in stereotactic treatments. Objectives were added to ensure that the prescription isodose closely conforms to the PTV and the dose to healthy lung tissue was minimized. The goal was to keep the fraction of healthy lung receiving more than 20 Gy (V_20 Gy_) below 10%. For the V_20 Gy_, both lungs minus the ITV were delineated, in accordance with the RTOG protocol. Maximum dose to the spinal cord was limited to 18 Gy, to the esophagus to 27 Gy, and to the heart to 30 Gy. To prevent the generation of very small segments the minimum beam segment area was set to 4 cm^2^, but generally the segment area was significantly larger. The minimum number of monitor units per segment was limited to 50 MU to ensure that the delivery time at least covers one breathing cycle. All plans consisted of 9 beams with in total generally 18 segments and in a few cases 17 segments.

Three different plans were created. The first treatment plan was optimized using full inhomogeneity corrections using the CC algorithm. A second plan was created assuming all tissues within the body to have unit density (UD), in accordance with the RTOG 0236 protocol. The third plan was optimized while only accounting for the decreased attenuation of the primary photons, thus resembling an equivalent pathlength (EPL) correction that is incorporated in less advanced dose calculation algorithms. These plans will be referred to as the CC, UD and EPL plans respectively. For all patients, these three plans were optimized until all planning criteria were met. A small renormalization was applied to all plans to ensure that they had exactly identical PTV coverage (60 Gy to 95% of the PTV). Next, the UD and EPL plans were copied and recalculated without re-optimization using the collapsed-cone algorithm.

For each plan the maximum, minimum and mean dose to the PTV was determined, as well as the dose received by 95% (D_95_) and 99% (D_99_) of the PTV, and the isocenter dose. Conformality of the PTV coverage was evaluated by the ratio of the volume of the prescription isodose (60 Gy) and the PTV (V_100%_/V_PTV_). For evaluation of the low dose spillage, the ratio of the 50% isodose volume (30 Gy) and the PTV was calculated (V_50%_/V_PTV_). The influence on lung dose was studied by scoring the lung volume receiving 20 Gy, 10 Gy (V_10 Gy_) and the mean lung dose (MLD). For all the other organs at risk, no further analysis was done as these received doses far below the tolerance dose by choosing appropriate beam arrangements. All differences in dosimetric parameters were tested using a paired-sample t-test.

## Results

### Planned dose distributions

**Figure 1:**
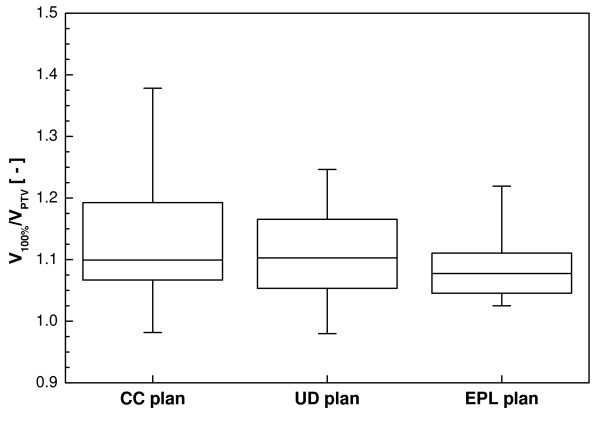
**Box-and-whisker plot of the conformity index for the optimized plan using full CC calculation, with unit density (UD) and with an equivalent pathlength (EPL) correction**.

For all three calculation algorithms, clinically acceptable treatment plans could be obtained for all patients (Table 2). The dose received by 99% of the PTV volume is slightly lower for the CC plans (58.5 Gy) compared to the UD (58.8 Gy) and EPL plans (58.8 Gy). Figure 1 shows a box-and-whisker plot of the ratio of the volume of the prescription isodose and the PTV, which is a measure for the conformity of a plan. The conformity for the UD and EPL plans was slightly better compared to the CC plans, although this difference was only statistically significant for the CC and EPL plans. The RTOG criterion (V_100%_/V_PTV _< 1.2) could however be met by all treatment plans except for 3 patients with very small tumors having a minor violation (V_100%_/V_PTV _< 1.4).

**Table 2:** Mean values and the range of dosimetric data for the different treatment plans as planned and recalculated using

	**CC**	**UD**				**EPL**			
	**Planned**	**Planned**	**p-value**	**Recalculated**	**p-value**	**Planned**	**p-value**	**recalculated**	**p-value**
D_95 _[Gy]	60.0	60.0	-	56.9 [42.1–63.4]	0.007	60.0	-	48.0 [34.6–56.1]	< 0.001
D_99 _[Gy]	58.5 [57.6–59.5]	58.8 [57.8–59.8]	< 0.001	54.5 [39.6–62.0]	< 0.001	58.8 [58.1–59.7]	< 0.001	45.9 [32.6–53.7]	< 0.001
D_isoc _[Gy]	74.3 [66.0–84.1]	66.6 [62.5–71.3]	< 0.001	72.7 [59.3–79.6]	0.19	67.1 [63.2–72.2]	< 0.001	61.7 [47.9–70.1]	< 0.001
D_mean _[Gy]	66.9 [64.1–71.0]	64.1 [61.6–66.6]	< 0.001	65.0 [49.1–71.0]	0.11	64.3 [61.8–66.4]	< 0.001	55.2 [39.7–62.1]	< 0.001
D_max _[Gy]	75.6 [67.8–87.4]	68.1 [63.0–73.3]	< 0.001	73.8 [61.3–81.7]	0.17	68.5 [63.8–76.5]	< 0.001	62.9 [48.0–74.2]	< 0.001
V_100%_/V_PTV_	1.13 [0.98–1.38]	1.09 [0.98–1.25]	0.14	0.98 [0.01–1.45]	0.08	1.09 [1.03–1.22]	0.026	0.27 [0.00–0.71]	< 0.001
V_50%_/V_PTV_	8.4 [4.5–18.2]	6.3 [4.4–12.6]	< 0.001	6.6 [4.9–12.2]	0.006	6.3 [4.1–12.0]	< 0.001	4.8 [3.1–6.5]	< 0.001
V_20 Gy _[%]	6.6 [2.9–16.7]	5.6 [2.2–14.2]	< 0.001	6.0 [2.3–15.5]	0.017	5.2 [2.1–12.3]	< 0.001	4.1 [1.5–10.9]	< 0.001
V_10 Gy _[%]	13.7 [7.3–35.8]	12.1 [5.9–29.9]	< 0.001	13.1 [6.1–33.5]	0.038	12.0 [5.5–29.5]	< 0.001	10.9 [4.6–27.3]	< 0.001
MLD [cGy]	456 [257–941]	396 [203–828]	< 0.001	439 [223–905]	0.18	394 [205–738]	< 0.001	355 [183–683]	< 0.001

full inhomogeneity corrections, and the p-values for the paired-sample t-test comparing the UD and EPL calculated and recalculated plans to the CC plan.

**Figure 2:**
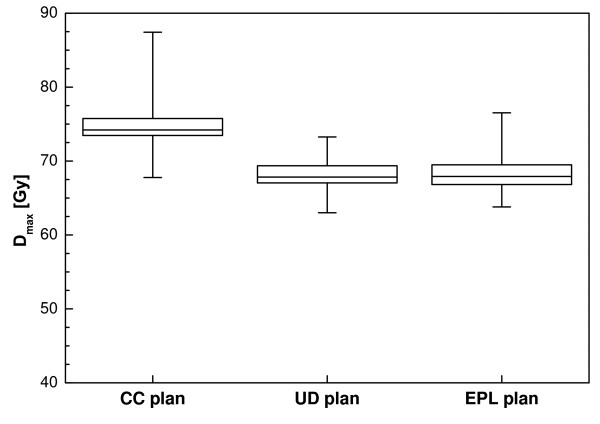
**Box-and-whisker plot of the maximum dose for the optimized plans before recalculation**.

**Figure 3:**
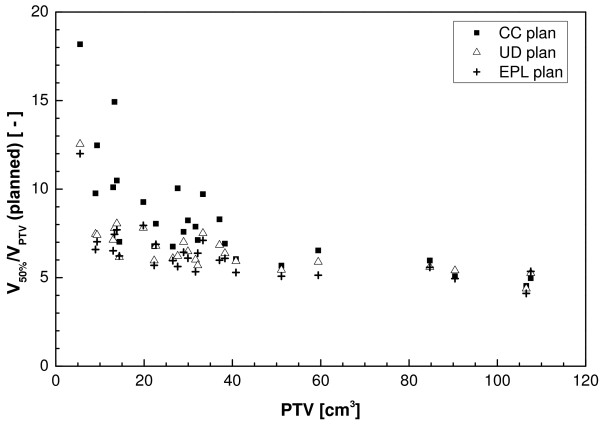
**Ratio of the volume of the 50% isodose and the PTV as a function of PTV for the three plans before recalculation**.

The maximum dose for the CC plans is considerably higher than for the other two (Figure 2). Dose homogeneity in the target area was less for the CC plans, as the effects of tissue inhomogeneities were better accounted for. The broadening of the dose distribution is reflected in the ratio of the volume of the 50% isodose and the PTV (V_50%_/V_PTV_) which is plotted as a function of the PTV in Figure 3. For the UD and EPL plans, significantly lower ratios were attainable (a mean value of 6.3 for both plans versus 8.4 for the CC plans). Figure 3 also shows that low-dose conformity decreased with decreasing PTV volume, especially for the CC plans.

Regarding the dose to the healthy lung tissue, the V_20 Gy _for the CC plans was on average 15% and 21% higher as the planned UD and EPL values, respectively (Table 2). For the mean lung dose, the UD and EPL plans both achieved a 13% lower value compared to the CC plan.

### Dose to target and critical organs after recalculation

**Figure 4:**
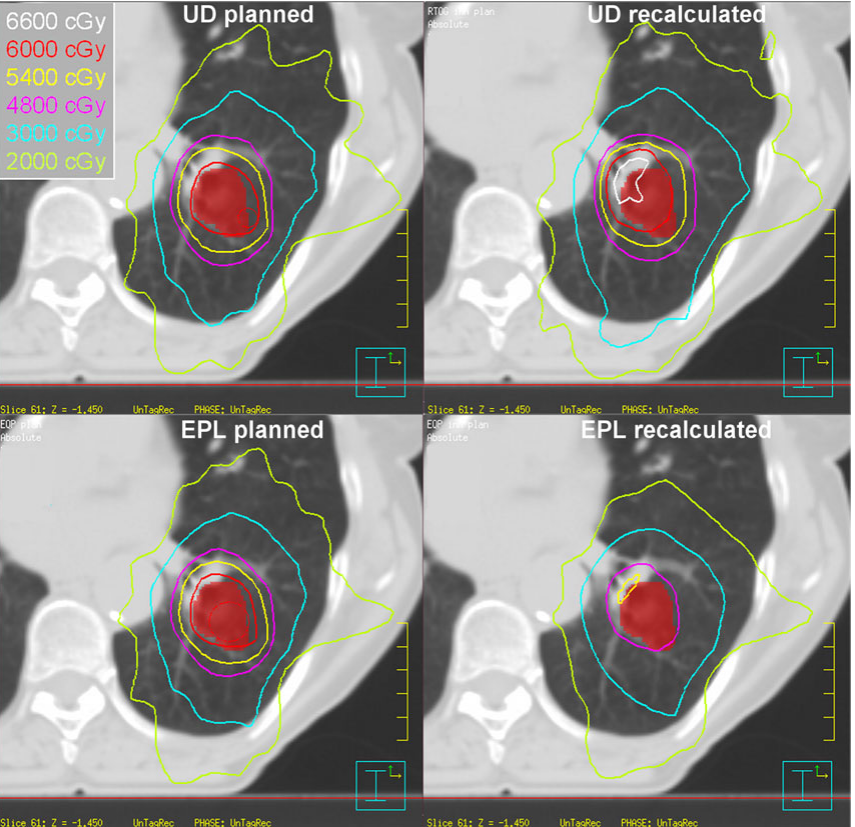
**Example of the planned and recalculated dose distribution for the UD (top) and EPL (bottom) calculation for a single patient**. Note the much higher dose to the hilar structure in the recalculated UD plan and the much lower dose coverage in the recalculated EPL plan.

The influence of recalculating the UD and EPL plans with the CC algorithm on the dose distribution for an individual patient is illustrated in Figure 4. The shape of the dose distribution changed significantly, leading to unwanted high dose regions, possibly endangering critical structures like the ribs or hilar vessels.

**Figure 5:**
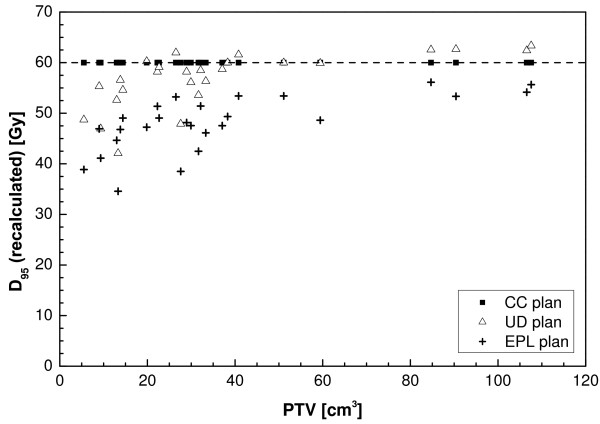
**Dose to 95% of the PTV as a function of the PTV as determined using the CC calculation**.

**Figure 6:**
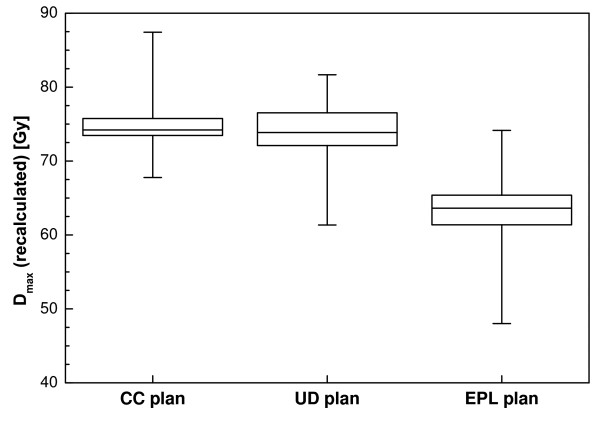
**Box-and-whisker plot of the maximum dose after recalculation of the unit density and EPL plan**. The results from the CC plan are plotted for comparison.

The dose covering 95% of the PTV for the recalculated plans as a function of the PTV is plotted in Figure 5. The EPL plans consistently overestimate the dose to the PTV, resulting in an average D_95 _of 48 Gy, 20% lower than the prescribed value. For one patient, a dose of 35 Gy was even observed, 43% lower than planned. The overestimation of the dose increased with decreasing PTV size, although large variations are observed between individual patients. For the UD calculations the recalculated plans on average had a slightly lower D_95 _of 57 Gy, with values ranging between 63 and 42 Gy for individual patients. No correlation was found with PTV size. For the other dosimetric parameters (D_99_, D_isoc_, D_mean_) similar results are found as for D_95 _(Table 2). For the maximum dose, larger values were found for the recalculated plans (Figure 6).

**Figure 7:**
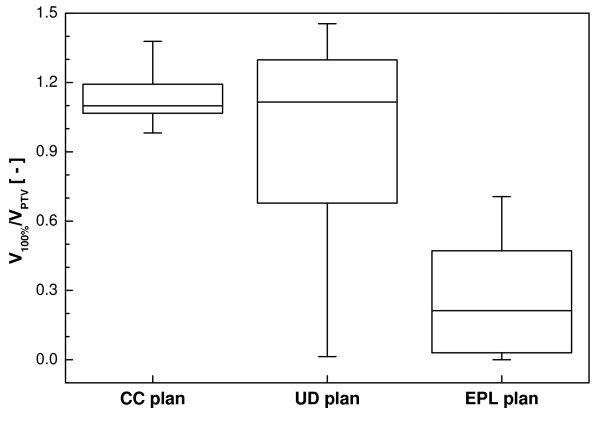
**Box-and-whisker plot of the conformity index for the UD and EPL plans after recalculation with full inhomogeneity corrections.** The results from the collapsed-cone calculation are plotted for comparison.

Changes in PTV coverage were also reflected in V_100%_/V_PTV _(Figure 7), with a significant decrease of this ratio for the EPL plans and also a small though not statistically significant decrease for the UD plans. Again, large variations between patients were especially visible for the UD plans, with ratios ranging from 0.01 to 1.45.

**Figure 8:**
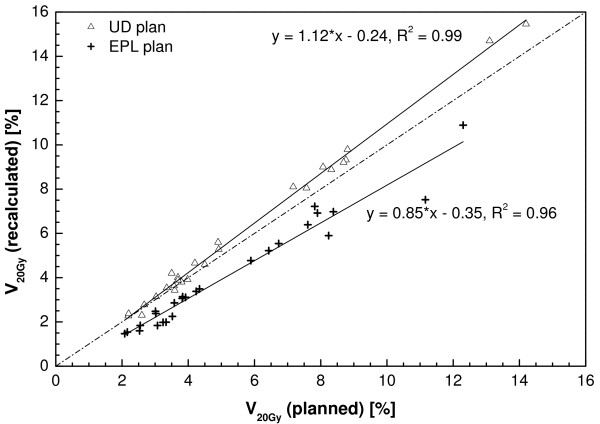
**Recalculated lung volume receiving 20 Gy or more versus planned value for the unit density and EPL plans**.

For the recalculated UD plans, the mean V_20 Gy _was significantly different from the CC plans (6.0 versus 6.6%), and large variations per patient existed. For the EPL plans the mean V_20 Gy _was significantly lower, with a mean value of 4.1%. The recalculated V_20 Gy _is plotted against the planned V_20 Gy _for the UD and EPL plans in Figure 8. These plots were fitted using linear regression, the resulting fit parameters can be found in table 3. A strong dependency existed between planned and recalculated values (R^2 ^= 0.99 and 0.96 for UD and EPL plans, respectively) although a reasonable amount of scatter is visible. For the V_10 Gy _and mean lung dose even stronger correlations were found between planned and recalculated values (Table 3). The recalculated V_10 Gy _was about 8% higher for the UD plans, and 8% lower for the EPL plans, for the MLD an 11% increase and 10% decrease was found, respectively.

**Table 3:** Correlation between planned and recalculated values for the lung volume receiving 20 Gy, 10 Gy and the mean lung dose.

	**Unit density**		**EPL**	
**Parameter**	**Correlation**	**R^2^-value**	**Correlation**	**R^2^-value**
V_20 Gy_	1.12·V_20 Gy, planned _- 0.24	0.994	0.85·V_20 Gy, planned _- 0.35	0.957
V_10 Gy_	1.08·V_10 Gy, planned _- 0.05	0.996	0.92·V_10 Gy, planned _- 0.07	0.979
MLD	1.11·MLD_planned _-	0.998	0.90·MLD_planned_	0.999

## Discussion

In this study it was demonstrated that the use of different inhomogeneity corrections during the planning of stereotactic lung RT treatments has a large impact on the dose distribution to the target area and healthy lung tissue. The separation into two types of algorithms (a and b) as mentioned in the introduction is of course a simplification of the differences that exist between the various clinically implemented algorithms. The comparison between the EPL and CC algorithms presented here can be seen as a good quantitative analysis of the differences that can be found between type a and b algorithms. However, slightly different results are expected if two other (implementations of) type a and b algorithms would have been used.

The dose criteria as prescribed in the RTOG 0236 trial [4] have been based on calculations assuming unit density of the patient. For the algorithms accounting for the effect of the increased lateral range of the electrons and scattered photons, these criteria are often harder to fulfill as the penumbras tend to be broadened.

Dose coverage of the recalculated plans varied widely among different patients. The dose to 95% of the PTV for the plans optimized with unit density ranged from 30% lower to slightly higher than planned for individual patients. A simple rescaling of the planned dose to the actual dose given to the patient is thus not possible, making a recalculation of the plan with accurate dose calculations necessary.

The overestimation of the dose using the EPL algorithm seen in all patients varies with increases tumorsize, lung density and location. This type of algorithm is still widely used in clinical practice, and has also been used by Lagerwaard *et al*. who recently presented clinical results of more than 200 patients with NSCLC [3]. The possible overestimation of the dose given to the tumor should be considered when interpreting the results from this clinical study.

In a study by Haedinger *et al*.[12] the stereotactic treatments from 33 lung cancer patients planned with a pencil beam algorithm were recalculated using a CC algorithm. These authors found a smaller overestimation of the dose given to the target using a pencil beam algorithm. This might in part be explained by the planning itself, as the PTV coverage in their work was often higher than the 95% used in this study. Another important difference is the PTV sizes considered in their study, with a median value of 122 cc compared to 29 cc in our study, which is more representative for patients suitable for this treatment. As a result smaller fieldsizes were used in our study, resulting in a larger overestimation of the dose.

As the UD and EPL calculations do not account for the increased lateral electron range, recalculation of the plans results in an increase of the low-dose region (V_20 Gy_, V_10 Gy_) in the lungs. On the other hand the algorithms often underestimate the required number of MUs due to lateral electron disequilibrium. As a result, the plans optimized with unit density calculations tend to underestimate the dose to the healthy lung (V_20 Gy_, V_10 Gy _and MLD), and the EPL plans overestimate it. In accordance with De Jaeger *et al*. [15] a correlation was found between planned and recalculated values for the lung dose. As this study deals with much smaller target volumes compared to the ones used by De Jaeger, lateral electron disequilibrium is more dominant, resulting in different correlations than the ones found by these authors. In contrast, Chang *et al *did not find a difference between V20 Gy values calculated for heterogeneity corrected and UD plans [16]. However, they predominately used simple anteroposterior/posterior-anterior fields and irradiated much larger lung volumes, as can be derived from their high V20 values in combination with their dose prescriptions. This again might explain the difference found between our results, using stereotactic radiotherapy to treat relatively small target volumes and the results derived by Chang *et al*. With yet another beam set-up, namely for breast irradiation, Brink *et al*. also found significant differences between algorithms in deriving optimal radiation pneumonitis NTCP values [17]. Thus, it might be concluded that differences between algorithms in calculating lung dose do exist. The extent of the deviation depends on the algorithms and irradiation techniques investigated.

Although Monte Carlo simulations are considered to be the gold standard in the presence of inhomogeneities, the collapsed cone algorithm has proven to be reasonably accurate. Krieger and Sauer did find up to 10% difference between CC and Monte-carlo calculations. However, these deviations were found using a slab geometry phantom and single beam set-up which does not resemble a clinical set-up very well. Furthermore, the authors indicate that part of these errors might be explained by an incorrect choice of the CC parameters [9]. In most situations, accuracy in the order of 2 to 5% is obtainable, meaning that inaccuracies in the CC dose calculations are much smaller than the differences observed in this study, and can be considered as a reasonably accurate representation of the actual dose given to the patient [18,17,10].

Maybe even more important, the collapsed cone algorithms have now become widely available in clinical practice, while the use of Monte Carlo treatment planning is still very limited. Thus, the CC results presented here can be used to generate optimization criteria in clinical practice, while this would be less straightforward for results based on Monte-Carlo calculations.

## Conclusion

The implications of the results in this study are twofold. In the first place, planning dose criteria are often easier achieved with plans created using simple dose calculation algorithms, which should be considered in study designs involving multiple institutions using different planning systems. Secondly, the actually delivered dose to the tumor can significantly deviate from the planned value when not using appropriate inhomogeneity corrections. As large variations exist in the actual dose per individual patient, clinical studies evaluating the effectiveness of this treatment should rely on the most accurate dose calculation which is clinically available at the time, or at least retrospectively re-evaluate the actual dose given to the target. Fortunately, the dose to the healthy lung tissue calculated with a simple algorithm can retrospectively easily be recalculated using the correlation parameters derived in this study. Before clinical introduction, the fractionation scheme and dose optimization procedure should be very well tailored to the calculation algorithm and TPS one uses clinically.

## Authors' contributions

DS was responsible for study design, carried out treatment planning, analysis of data and results, and writing and editing the manuscript, CWH worked on study design, analysis of data and results, and writing and editing the manuscript. All authors have read and approved the final manuscript.
